# The impact of acute stress on athletes’ perceptions of fairness in decision-making and its neural mechanisms

**DOI:** 10.3389/fnhum.2025.1685000

**Published:** 2025-10-30

**Authors:** Jiayuan Ji, Huiling Wang, Shiyu Wang, Yutong Ye, Yitong Zhang, Lin Li

**Affiliations:** ^1^School of Physical Education and Health, East China Normal University, Shanghai, China; ^2^Key Laboratory of Adolescent Health Evaluation and Exercise Intervention, Ministry of Education, East China Normal University, Shanghai, China; ^3^Institute of Physical Education, Xi’an Jiaotong University, Xi’an, China

**Keywords:** acute stress, athletes, sense of unfairness decision-making, neural mechanisms, functional near-infrared spectroscopy (fNIRS)

## Abstract

**Background:**

Acute stress may disrupt decision - making by affecting cognitive and emotional processing. The behavioral and neural mechanisms of this in athletes are unclear. This study explored how acute stress impacts athletes’ unfairness - related decision - making and its neural basis.

**Methods:**

Forty participants (20 university athletes and 20 non-athletes) were randomly assigned to a stress group or a control group. Using functional near-infrared spectroscopy (fNIRS), the study monitored the prefrontal cortex (PFC) and temporoparietal junction (TPJ) blood oxygenation during an ultimatum game task after inducing acute stress via the Maastricht Acute Stress Test (MAST).

**Results:**

Athletes under stress were more accepting of relatively unfair decisions than non-athletes. This was linked to lower activation in the frontal-eye areas (CH15), supramarginal gyrus (CH38), and somatosensory association cortex (CH67), and higher activation in the primary motor cortex (CH64) in athletes. The increase in acceptance efficiency correlated significantly with the reduced CH38 activation (*r* = −0.425) and increased CH64 activation (*r* = 0.499).

**Conclusion:**

Long-term exercise training may promote athletes’ tendency to accept relatively unfair decisions under acute stress by modulating activation levels in the supramarginal gyrus and primary motor cortex, demonstrating stronger adaptive behavior. These findings offer insights for developing stress management and neuromodulation training programs for athletes.

## 1 Introduction

Fairness perception is one of the core topics in human behavior research. When individuals perceive a violation of fairness principles, it may trigger irrational behavior (Guo et al., 2013; Sanfey et al., 2003) or negative responses (Halali et al., 2014). Decisions based on subjective perceptions of fairness are termed inequity decisions, commonly measured through the Ultimatum Game (UG) and the Dictator Game (DG). In competitive sports, athletes frequently encounter acute stressors such as personal performance errors, sudden weather changes, or unexpected injuries. Under such stress, their judgments of fairness regarding referees’ decisions or opponents’ provocations may elicit irrational reactions, which could impair individual performance and even team outcomes. Consequently, investigating athletes’ perceptions of unfairness and decision-making under acute stress may help optimize their coping strategies, reduce stress-induced decision risks, and provide valuable implications for competitive sports practice.

Existing research has shown that acute stress can influence unfairness-related decision-making. For instance, Cano-López et al. (2016) reported that individuals under acute stress are more likely to reject unfair offers. A tendency thought to be associated with stress-induced emotional fluctuations such as anger or anxiety, which heighten sensitivity to unfairness (Brüne et al., 2013; van’t Wout et al., 2010). However, opposite findings have also been reported, Takahashi et al. (2007) using the Dictator Game (DG), observed that individuals became more generous in accepting unfair offers following acute stress. According to the cross-stressor adaptation hypothesis, prolonged high-intensity training may induce beneficial adaptations in stress-response systems, enabling individuals to cope with acute stress more efficiently (Sothmann et al., 1996). This suggests that decision-making behavior under stress conditions may differ between athletes and the general population. Supporting this notion, prior studies have shown that volleyball athletes exhibited decreased accuracy in cognitive decisions but improved accuracy in intuitive decisions following acute stress (Xie, 2023); Similarly, high-level athletes demonstrated greater decision accuracy under high arousal, whereas lower-level athletes performed better under low arousal (Luna, 2017). Nevertheless, direct empirical evidence regarding the impact of acute stress on unfairness-related decision-making in athletes remains scarce, and the underlying neural mechanisms require further investigation.

Recent advances in brain function research have provided important neural insights into the relationship between acute stress and decision-making. Networks such as the default mode network (DMN) and the central executive network (CEN) have been shown to be involved in acute stress responses (Hermans et al., 2011, 2014; Qin et al., 2009; Vaisvaser et al., 2013). Key regions include the medial prefrontal cortex (mPFC), inferior parietal lobule (IPL), dorsolateral prefrontal cortex (dlPFC), dorsomedial prefrontal cortex (dmPFC), and frontal eye field (FEF) (van Oort et al., 2017). Furthermore, studies have revealed that acute stress primarily activates the prefrontal cortex (PFC) (McEwen, 2007; Rodrigues et al., 2009) and the temporoparietal junction (TPJ) (Li et al., 2023). Researchers have found that acute stress interferes with prefrontal cortex function through activation of the Hypothalamic-Pituitary-Adrenal Axis (HPA axis) and the sympathetic nervous system (Furay et al., 2008; Kern et al., 2008), leading to decreased inhibitory control and emotion regulation (Kern et al., 2008; McEwen, 2007), which in turn affects fairness judgment (Peng and Zhou, 2007; Qiu et al., 2013). For example, a transcranial magnetic stimulation (TMS) study demonstrated that inhibition of the right dlPFC increased individuals’ tendency to accept unfair offers (Baumgartner et al., 2011), suggesting that changes in dorsolateral prefrontal functioning may be an acute stress affecting decision-making. Meanwhile, growing evidence indicates that long-term exercise exerts profound effects on cognition, emotion, and brain function. Regular physical training has been shown to strengthen functional connectivity within the prefrontal–striatal circuit, thereby enhancing cognitive control and emotional regulation (Faubert, 2013). Moreover, exercise has been found to mitigate stress-induced neural damage in the amygdala and hippocampus, promoting faster recovery (Micheli et al., 2018; Wang et al., 2013). These findings suggest that exercise experience may induce neuroplastic changes that reshape neural regulation. However, whether such exercise-induced plasticity can buffer the impact of stress on decision-making remains an open question.

To elucidate how athletes’ unfairness-related decision-making is influenced by acute stress and to uncover the underlying neural mechanisms, this study employed functional near-infrared spectroscopy (fNIRS) to monitor hemodynamic responses in PFC and TPJ of athletes and ordinary university students while performing the UG following acute stress induction. Using a randomized controlled design, we hypothesize that acute stress alters athletes’ responses to unfair offers and modulates activation patterns in the PFC and TPJ. The findings are expected to provide empirical evidence for optimizing psychological regulation and stress management strategies in athletes, thereby enhancing decision-making efficiency and competitive performance, while also contributing to the refinement of stress-related decision-making models and broadening the scope of research in sports neuroscience.

## 2 Materials and methods

### 2.1 Participants

To determine the required sample size, a priori power analysis was conducted using G*Power 3.1 (Faul et al., 2009). Given the four (groups: athlete stress group; athlete sitting group; non-athlete stress group; non-athlete sitting group) × 2 (time: pre-test; post-test) mixed experimental design, an *F*-test for ANOVA was selected. A medium effect size (*f* = 0.30) was specified, with statistical power (1−β) set at 0.80, indicating an 80% probability of correctly rejecting a false null hypothesis. The significance level (α) was set at 0.05, reflecting a 5% risk of rejecting a true null hypothesis. Based on these parameters, the required sample size was calculated to be 36 for this experiment.

A total of 40 participants were recruited from a university in Shanghai, comprising 20 student-athletes and 20 non-athletes, with an equal sex distribution. Participants were randomly assigned to one of four groups, with 10 individuals per group (balanced by sex), and with the ages ranging from 17 to 24 years old. The athlete group consisted of individuals with at least 5 years of systematic training experience across sports such as basketball, soccer, and track and field. Each group consists of five athletes, including three national first-level athletes and two national second-level athletes. Non-athletes had no history of systematic sports training. All participants were right-handed, had normal or corrected-to-normal vision, and reported no history of color blindness, psychiatric disorders, or neurological conditions. The study protocol adhered to the ethical standards of the Declaration of Helsinki and was approved by the Human Research Ethics Committee of East China Normal University (approval number: HR2-0125-2025). Written informed consent was obtained prior to participation, and participants received monetary compensation upon completion of the study.

### 2.2 Experimental procedure

**FIGURE 1:**
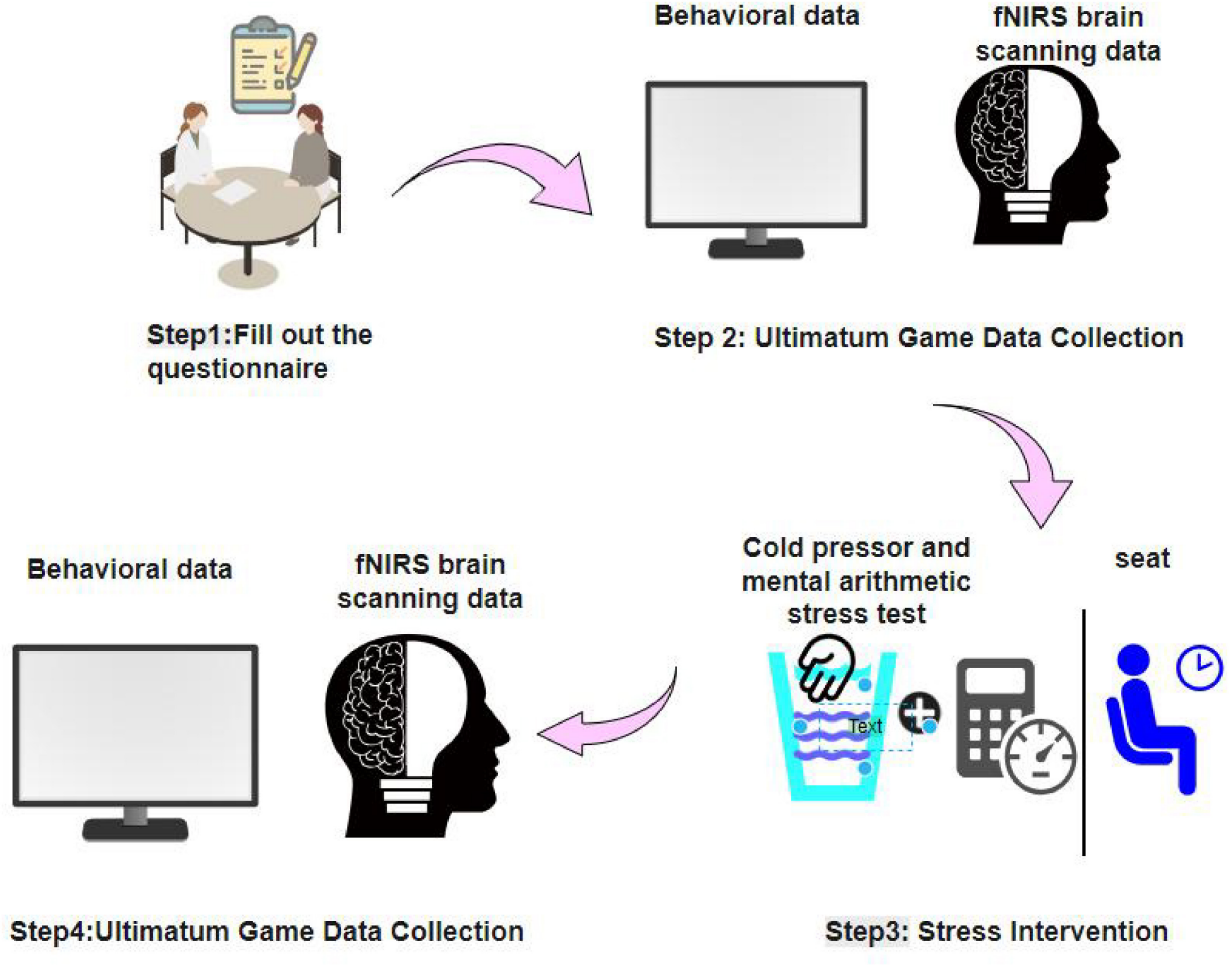
Experimental flowchart.

Upon arrival at the laboratory, participants first read the informed consent form. After fully understanding the study procedures and agreeing to participate, they provided written informed consent. The experimenter then gave a detailed explanation of the procedure to ensure that each participant was familiar with all stages of the study. Prior to the experimental tasks, participants completed a demographic questionnaire and additional self-report scales, including Colquitt’s Organizational Justice Scale (OJS), Chinese Five Personality Scale 2018 (CFPS-2018), Sense of Power Scale (SPS), Risk Attitude Scale (RAS), and Depression Anxiety Stress Scales - 21 (DASS-21). At the start of the experiment, participants were seated in front of a computer and fitted with an fNIRS cap, a heart rate monitor, and a blood pressure cuff. They then entered a 3-min resting state during which baseline heart rate and blood pressure were recorded. Following the rest period, participants performed the first UG task. Subsequently, participants assigned to the stress condition underwent the Maastricht Acute Stress Test (MAST), which combines cold pressor and mental arithmetic tasks, with direct video monitoring to enhance stress induction. Participants in the control condition engaged in a 4-min seated rest. Throughout both conditions, heart rate and blood pressure were continuously recorded. Finally, all participants performed a second UG task while seated at the computer. The overall experimental procedure is illustrated in Figure 1.

### 2.3 The Maastricht Acute Stress Test, MAST

To induce acute stress, this study employed a modified version of the Maastricht Acute Stress Test (Smeets et al., 2008, 2012), consisting of alternating cold pressor and mental arithmetic tasks with a total duration of 4 min. In the cold pressor task, participants immersed their left hand in ice water maintained at 0 °C–4 °C for 1 min. Immediately afterward, they removed their hand, placed it on a towel on the desk, and completed the mental arithmetic task (e.g., serial subtraction of 17 from 2043) for 1 min. Each cycle of cold pressor plus arithmetic lasted 2 min, and participants completed two consecutive cycles. Throughout the procedure, a video camera was positioned directly in front of the participant, and they were informed that their facial expressions would be continuously recorded.

### 2.4 Ultimatum game

This experiment used an ultimatum game task (Güth et al., 1982) prepared by E-Prime 2.0. In the task, the participants to be observed by the experiment acted as responders and made decisions interactively with the virtual proposer via a computer monitor. There were three allocation scenarios (extremely unfair: 1:29–5:25; relatively unfair:10:20–14:16; and absolutely fair:15:15), and each type of scenario was presented 10 times each after pseudo-randomization (total trials = 30). To enhance ecological validity, three practice rounds are conducted before the formal experiment, and participants are clearly informed in the task instructions that the amount earned from their decisions will be proportionally converted into additional cash rewards and distributed after the experiment.

**FIGURE 2:**
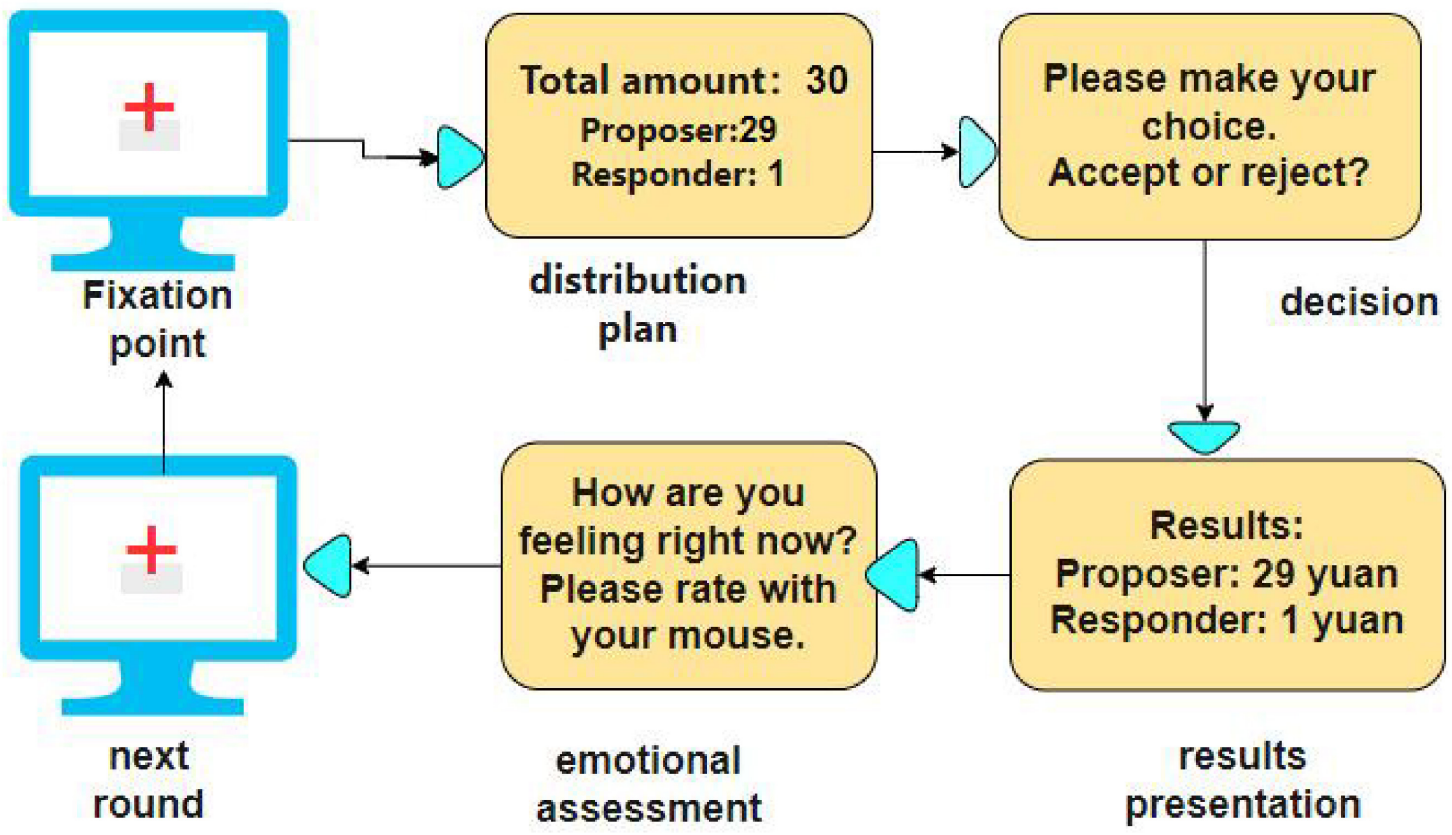
Task flowchart.

The flow of the experiment is shown in Figure 2. First, a “+” gaze point appeared on the screen for 2 s. Then the allocation scheme was displayed on the screen for 4 s. Participants were required to make a decision by pressing a button (F/J) to accept or reject the proposal within 10 s. Subsequently, the results of the choice, including the participant’s in-game income and the virtual proposer’s choice, were displayed for 4 s. This was followed by a mood assessment phase, in which participants rated their current mood (“very sad” = 1 to “very happy” = 9) via a button press within 4 s. At the end, the “†” gaze point screen appeared again, signaling the start of the next round. Each round takes about 25 s to present and lasts about 13 min in total.

### 2.5 Measurement questionnaires

Previous studies have demonstrated that perceptions of unfairness are influenced by various social factors, including sex, personality, and emotional states (Youssef et al., 2018; Fang et al., 2021; Tang, 2019). The present study employed a series of standardized questionnaires to assess and control for these variables. These questionnaires, used as the additional self-reports in see section “2.2 Experimental procedure,” are detailed below to provide a comprehensive understanding of their design and purpose.

#### 2.5.1 Colquitt’s Organizational Justice Scale (OJS)

Based on Colquitt et al. (2001), Colquitt’s Organizational Justice Scale containing four dimensions of procedural justice (7 items), distributive justice (4 items), interpersonal justice (4 items), and informational justice (5 items), scored on a five-point scale (1 = never, 5 = frequently), with higher total scores indicating fewer experiences of unfairness.

#### 2.5.2 Chinese five personality scale 2018 (CFPS-2018)

The CFPS-2018 (Wu and Liping, 2020) has five dimensions, and its design is based on the Five - Factor Model (FFM), which measures the personality trait scores of dutifulness, extroversion, affinity, openness, and emotional instability, respectively. The questionnaire consists of 15 questions, with 1–5 scoring options, and the total dimension score reflects the strength of the trait, with higher scores being more prominent.

#### 2.5.3 Sense of power scale (SPS)

Developed by Anderson et al. (2012), the SPS assesses individuals’ perceived sense of power in social interactions. Participants rate each item on a seven-point scale (1 = strongly disagree, 7 = strongly agree), with higher scores reflecting stronger perceived power.

#### 2.5.4 Risk attitude scale

The RAS Developed by Weber et al. (2002) evaluates risk-taking tendencies across six domains. It captures individuals’ risk attitudes and decision-making preferences through domain-specific scenarios, providing a multidimensional profile of risk perception and acceptance.

#### 2.5.5 Depression anxiety stress scales - 21 (DASS-21)

The DASS-21 (Lovibond and Lovibond, 1995) is a widely used instrument for assessing depression, anxiety, and stress. Adapted from the original 42-item version, the DASS-21 contains 21 items across three subscales (seven items each for depression, anxiety, and stress). Items are rated on a four-point scale, with higher scores indicating greater symptom severity. Each entry is scored according to how often or how severely the subject has experienced symptoms in the past week, ranging from 0 (never) to 3 (almost always). The final subscale scores involve adding the seven entry scores and multiplying by 2 (range 0–42) and categorizing the severity according to the following criteria: depression (0–9 normal, 10–13 mild, 14–20 moderate, 21–27 severe, 28 + very severe), anxiety (0–7 normal, 8–9 mild, 10–14 moderate, 15–19 severe, 20 + very severe), stress (0–14 normal, 15–18 mild, 19–25 moderate, 26-33 severe, 34 + very severe).

### 2.6 Data collection and processing

#### 2.6.1 Behavioral data collection and processing

In this study, the behavioral data included questionnaire data, participants’ heart rate and blood pressure, reaction time, choice preferences (e.g., risky or conservative choices) in the ultimatum gaming game. The data were quantitatively analyzed by the experimenters, and these data can help to understand how stress affects the decision-making process, especially how athletes make immediate decisions in stressful situations.

#### 2.6.2 Brain data acquisition and processing

**FIGURE 3:**
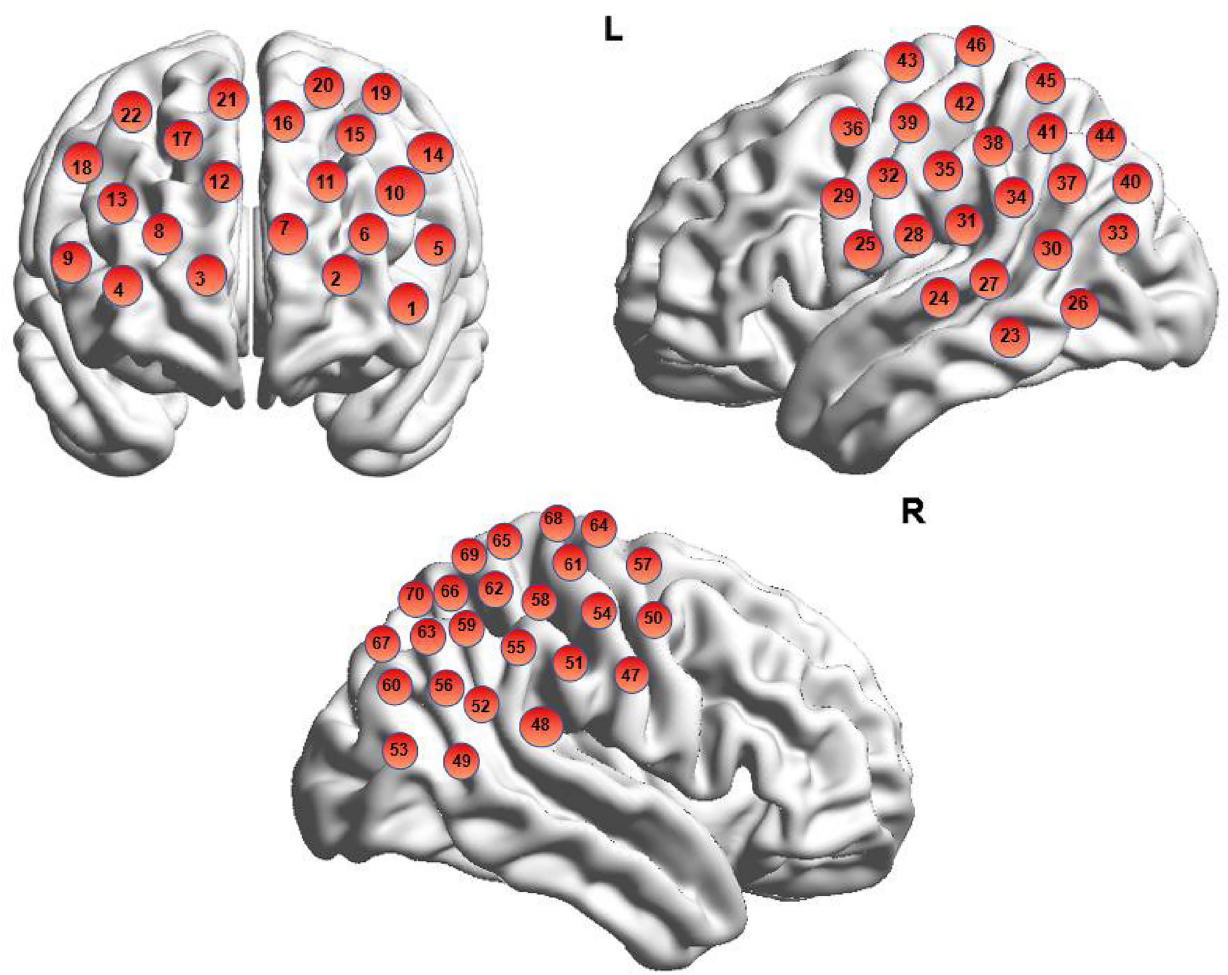
Schematic diagram of brain channel localization.

In this study, a Hitachi ETG-7100 near-infrared spectroscopic imaging system (Hitachi Medical Corporation, Japan) was used to continuously monitor functional near-infrared spectroscopy (fNIRS) data. The system operated at wavelengths of 695 and 830 nm, with a sampling frequency of 10 Hz, and a probe array covering a 3 × 5 channel layout (70 channels in total) centered on Fpz (International 10–20 System), with a distance of 3 cm between probes. The device records signals of changes in blood oxygenation in the brain during the task. To determine the cortical regions underlying each channel, this study applied a widely adopted virtual spatial registration method (Tsuzuki et al., 2007). A 3D digitizer was first used to calibrate probe positions by sequentially marking five anatomical landmarks (Nz, Cz, Lz, AL, AR), as well as the emitter, detector, and channel locations on the scalp. These coordinates were then mapped onto the Montreal Neurological Institute (MNI) standard brain using NIRS-SPM, allowing anatomical labeling based on the Automated Anatomical Labeling (AAL) atlas and Brodmann areas (Singh et al., 2005) (see Supplementary Table 6). The regions of interest in this study included the prefrontal cortex (PFC) and bilateral temporoparietal junctions (TPJ). Spatial registration accuracy and potential resolution were further validated using the maximum probability method. Channel localization results are illustrated in Figure 3, with detailed anatomical coordinates provided in the Supplementary material. This study used BrainNet Viewer (Xia et al., 2013) for brain visualization.

Existing studies have shown that oxyhemoglobin (Hbo) is more sensitive to task-related stimuli (Rahman et al., 2020), so this study used MATLAB to observe only the changes in Hbo.

Raw fNIRS data, in addition to the neural activity elicited by the executive function tasks of interest, also contain noise from the instrument, physiological interference from respiration and heartbeat, and motion artifacts. To obtain more accurate and smoother data, preprocessing is performed using the NIRS-SPM toolkit (Ye et al., 2009), which operates based on a linear regression model on the MATLAB R2014a platform. This involves steps such as data conversion, time-series correction, filtering, and noise reduction.

First, the modified Lambert-Beer law is applied to convert the raw optical density signals into blood oxygen concentration data (Cope et al., 1988). Next, filtering is employed to eliminate interference signals caused by noise and physiological factors. All near-infrared data are filtered using a low-pass filter based on hemodynamic response functions (HRF) to attenuate high-frequency non-neuronal components (Brigadoi et al., 2014) and a high-pass filter based on discrete cosine transform (DCT) to remove pseudo-noise caused by activities such as heartbeat and respiration (Jang et al., 2009). Subsequently, the general linear model (GLM) is used to derive beta values for the task state, reflecting and evaluating cortical activation patterns throughout the task (Plichta et al., 2007a,b). Finally, differential tests are conducted on the beta values obtained from different groups. All *p*-values are corrected using the false discovery rate (FDR) method, with a significance threshold of *p* < 0.05. *T*-value heatmaps are generated and mapped onto a 3D brain model using the BrainNet Viewer toolkit to visualize blood oxygen level changes in different brain regions under varying decision-making conditions.

To ensure data quality and the reliability of the analysis results, statistical testing methods are applied during preprocessing to remove anomalous data caused by motion or equipment malfunctions. Specifically, if the standard deviation of the GLM residuals for a given channel exceeds three times the overall residual standard deviation, that channel is flagged as an outlier and removed.

### 2.7 Data validation and analysis

The data were analyzed in this study using IBM SPSS 23.0. First, demographic information and questionnaire data were analyzed by one-way analysis of variance (ANOVA) with group as a factor to examine differences between groups. Next, heart rate and blood pressure data were subjected to a four (groups: athlete stress group; athlete sitting group; non-athlete stress group; non-athlete sitting group) × 2 (time: pre-test; post-test) repeated measures ANOVA to test for indicators of significant main and interaction effects. Next, the behavioral data were subjectd to a four (groups: athlete stress group; athlete sitting group; non-athlete stress group; non-athlete sitting group) × 2 (time: pre-test; post-test) repeated-measures ANOVA with post-hoc tests for significant indicators and tests for interaction effects (performing Bonferroni correction). The fNIRS data were then subjected to a two (groups: athlete; non-athlete) × 2 (condition: stress;sitting) × 2 (time: pre-test; post-test) multivariate ANOVA based on the behavioral results, with further analyses of the channels where interactions and main effects were present. Finally, Spearman correlation analysis was used to examine the relationship between behavioral changes and brain activation changes. Bootstrap sampling (1,000 iterations, 95% confidence interval) was applied to assess the robustness and stability of the correlation coefficient, accounting for potential variability in the data distribution to ensure a reliable estimation of the confidence interval for the correlation.

## 3 Results

### 3.1 Descriptive statistics

In order to explore the differences between the different groups on age, level of perceived fairness, experience of sense of power, and three-dimensional mood indicators of depression, anxiety, and stress. A one-way ANOVA was performed on the pre-test data, and it was found that there were no significant differences in any of the indicators (see Table 1).

**TABLE 1:** Descriptive statistics.

Indicator	Group	Mean	Standard deviation	F	*P*	η^2^
Age (years)	1	18.80	0.63	1.14	0.346	0.03
	2	18.80	1.14			
	3	19.80	2.30			
	4	18.70	1.57			
Years of training (years)	1	8.20	3.68	1.29	0.212	0.30
	2	5.70	4.88			
	3	0.30	0.95	0.74	0.470	0.18
	4	0.08	0.17			
Sense of fairness	1	43.50	7.11	1.41	0.257	0.04
	2	46.50	6.38			
	3	45.60	7.92			
	4	49.60	5.36			
Personality	1	47.50	8.33	1.88	0.151	0.05
	2	47.60	4.84			
	3	50.10	4.58			
	4	56.50	16.33			
General powers	1	30.50	2.80	0.61	0.616	0.01
	2	28.10	4.31			
	3	29.20	6.22			
	4	30.10	3.18			
Risk assessment	1	21.70	4.42	.95	.427	0.02
	2	20.30	3.16			
	3	23.10	4.95			
	4	23.00	4.32			
Pressure	1	6.50	4.40	0.22	0.886	0.01
	2	7.60	3.03			
	3	8.40	7.93			
	4	7.10	5.38			
Anxiety	1	8.90	7.28	0.90	0.451	0.03
	2	5.70	5.38			
	3	6.80	3.46			
	4	5.50	3.78			
Depression	1	7.40	6.93	1.57	0.214	0.04
	2	4.20	3.05			
	3	5.40	4.99			
	4	2.90	3.32			

1, athlete stress group; 2, athlete sitting group; 3, non-athlete stress group; 4, non-athlete sitting group.

### 3.2 Effects of acute stress on blood pressure and heart rate

To investigate the effects of acute stress on blood pressure and heart rate, a repeated measures analysis of variance was conducted with a four (Groups: athlete stress group; athlete sitting group; non-athlete stress group; non-athlete sitting group) × 2 (time: pre-test; post-test) design. After Bonferroni correction, the results showed that in terms of blood pressure, there were no significant differences in systolic or diastolic blood pressure among the four groups during the pre-test phase. In the post-test phase, significant differences were observed between the athlete stress group and the athlete sedentary group in both systolic (p_sys = 0.006) and diastolic blood pressure (p_dia = 0.042). Post-stress, athletes exhibited a significant increase in systolic blood pressure (*p* = 0.020), while the difference in diastolic blood pressure was not significant (*p* = 0.116). In contrast, no significant changes in systolic or diastolic blood pressure were observed in ordinary college students before and after stress. Regarding heart rate, no significant differences were observed either between groups or before and after stress. The significant changes in blood pressure suggest that the effects of acute stress are valid for both athletes and the average college student (see Supplementary Figure 1 and Supplementary Table 1).

### 3.3 Effects of acute stress on athletes’ unfair decision-making behavior

To investigate the impact of acute stress on unfair decision-making behavior, a 4(Groups: athlete stress group; athlete sitting group; non-athlete stress group; non-athlete sitting group) × 2 (Time: pre-test; post-test) repeated measures ANOVA was conducted for the C1 extremely unfair scenario, C2 relatively unfair scenario, and C3 absolutely fair scenario (see Supplementary Table 2). Results revealed significant main effects of time across all three conditions (*p*_*C*1–*reject*_ < 0.001, η^2^ = 0.32; *p*_*C*2–*reject*_ < 0.001, η^2^ = 0.33; *p*_*C*2–*accept*_ = 0.005, η^2^ = 0.20; *p*_*C*3–*accept*_ < 0.001, η^2^ = 0.48), the main effect of group for the relatively unfair condition (*p*_*C*2–*reject*_ < 0.001, η^2^ = 0.39; *p*_*C*2–*accept*_ = 0.001, η^2^ = 0.38) and the interaction effect were significant (*p*_*C*2–*reject*_ = 0.002, η^2^ = 0.34; *p*_*C*2–*accept*_ = 0.033, η^2^ = 0.21) (see Supplementary Table 3).

To compare the differences between athletes and regular college students in their decision-making on the sense of unfairness, a post-hoc test on the pre-test data found that there were no significant differences between athletes and regular college students in the rejection and acceptance efficiencies of the three scenarios (*p*_*C*1–*reject*_ = 0.397; *p*_*C*1–*accept*_ = 0.751; *p*_*C*2–*reject*_ = 0.054; *p*_*C*2–*accept*_ = 0.248; *p*_*C*3–*reject*_ = 0.417; *p*_*C*3–*accept*_ = 0.138), indicating that athletes and regular college students make the same decisions about feelings of unfairness.

**FIGURE 4:**
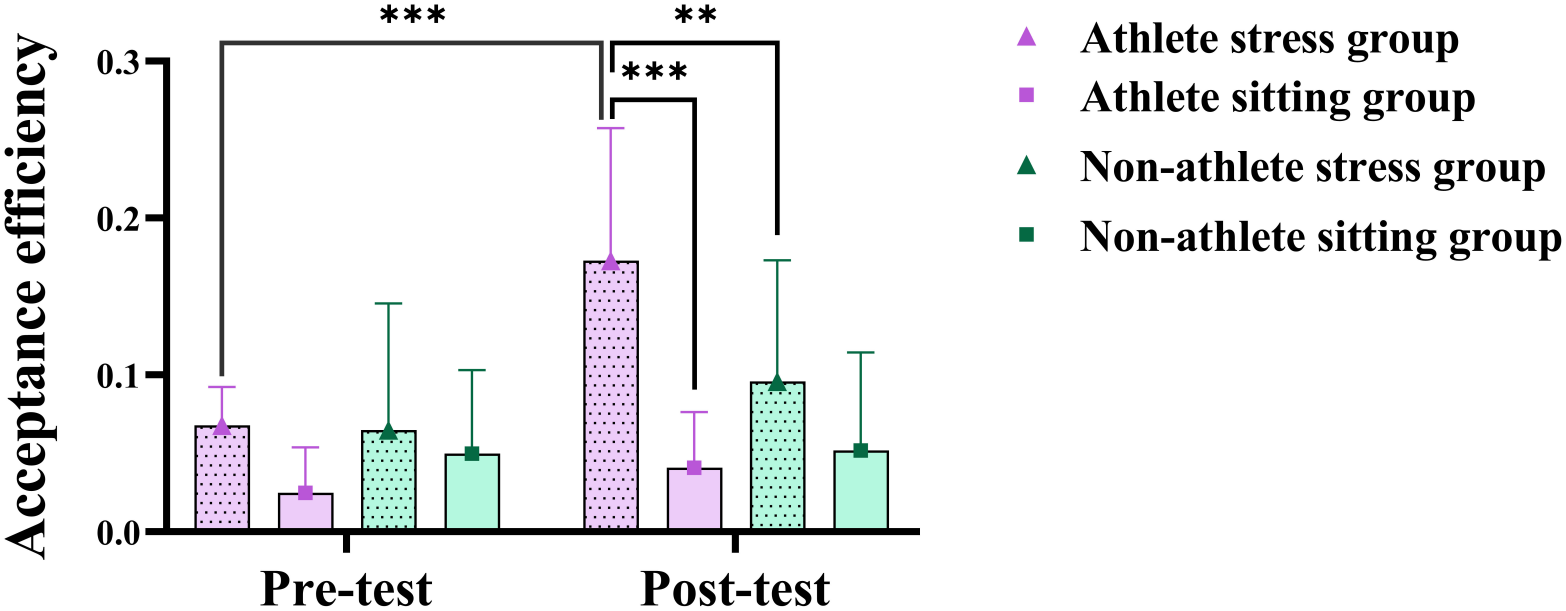
Three-way analysis of variance (ANOVA) results of acute stress on relatively unfair decision-making behavior. The symbols denote significance levels: *represents *p* < 0.05, **represents *p* < 0.01, ***represents *p* < 0.001.

To investigate acute stress’s impact on athletes’ unfairness decision-making, simple effects analysis was conducted on the relative unfairness scenario exhibiting interaction effects. Results revealed that the post-test acceptance efficiency in the athletes’ stress group was significantly higher than that in the athletes’ sitting group (*p* < 0.001, Cohen’s *d* = 0.46) and the general university students’ stress group (*p* < 0.01, Cohen’s *d* = 0.79), and significantly higher than the pre-test (*p* < 0.001,Cohen’s *d* = 0.82) (see Figure 4). On the rejection efficiency, the difference between the pre-test and post-test of the athletes’ stress group was not significant (*p* > 0.05). The above results indicate that acute stress can significantly affect athletes’ decision-making on the sense of relative unfairness, and athletes will be more inclined to make the decision of accepting the relative unfairness programme after stress compared with non-athletes.

### 3.4 Effects of acute stress on brain activation during athletes’ acceptance of relatively unfair proposals

**FIGURE 5:**
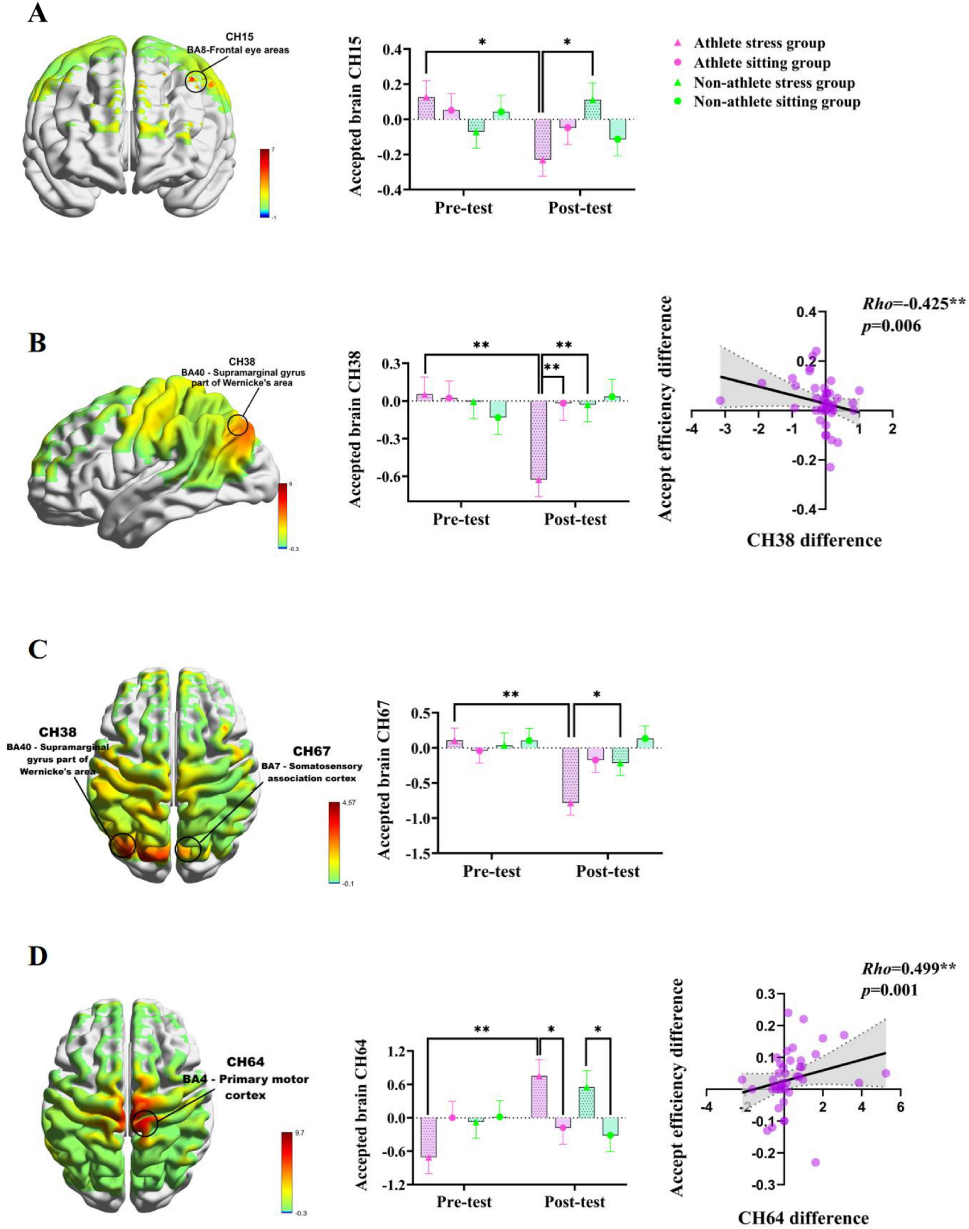
**(A)** Brain mapping of the *F*-value for the Group × Condition × Time interaction effect and specific changes in CH15; **(B)** Group × Time interaction effect *F*-value brain mapping and specific changes in CH38 and correlation between CH38 changes and acceptance efficiency changes; **(C)** Group × Time interaction effect *F*-value brain mapping and specific changes in CH67; **(D)** Condition × Time interaction effect *F*-value brain mapping and specific changes in CH64 and correlation between CH64 changes and acceptance efficiency changes. The symbols denote significance levels: *represents *p* < 0.05, **represents *p* < 0.01.

To investigate the neural mechanisms underlying acute stress’s influence on athletes’ acceptance of relatively unfair proposals, a three-factor mixed design [2 (Groups: athlete; non-athlete) × 2 (Condition: stress;sitting) × 2 (Time: pre-test; post-test)] was employed (see Supplementary Tables 4, 5). The ANOVA results revealed a significant interaction effect for the group × condition × time (*p* = 0.011, η^2^ = 0.086). Further post-hoc tests revealed that activation on the post-test was significantly lower than on the pre-test in the athlete stress group (*p* = 0.009) and significantly lower than on the post-test in the stress group of the average college student (*p* = 0.013); whereas activation on the post-test was significantly lower than on the pre-test in the sitting group of the average college student (*p* = 0.017) (see Figure 5A).

At CH38 (located in the supramarginal gyrus, Brodmann area 40), the interaction effects of group × time (*p* = 0.028, η^2^ = 0.065) and Condition × Time (*p* = 0.035, η^2^ = 0.06) interaction effects were significant. Further *post hoc* analyses revealed that athletes in the stress group exhibited significantly lower activation at post-test compared to pre-test (*p* = 0.001), and significantly lower than both the post-test levels of the exercise-meditation group (*p* = 0.002) and the stress group of non-athletes (*p* = 0.003) (see Figure 5B). At CH67 (located in the Somatosensory Association Cortex, Brodmann area 7), the group × time interaction was significant (*p* = 0.035, η^2^ = 0.06). *Post hoc* analysis revealed that post-test activation in the athlete stress group was significantly lower than pre-test (*p* = 0.001) and significantly lower than the post-test in the general student stress group (*p* = 0.025) (see Figure 5C). At CH64 (located in the Primary Motor Cortex, M1, Brodmann area 4), the condition × time interaction effect was significant (*p* = 0.003, η^2^ = 0.12). Further post-hoc tests revealed that activation was significantly higher in the athlete stress group post-test than in the pre-test (*p* = 0.001) and significantly higher than the post-test in athlete sitting group (*p* = 0.028); Meanwhile, the post-test activation in the stressed group of non-athletes was significantly higher than that in the post-test of the seated group of non-athletes (*p* = 0.040) (see Figure 5D). These results indicate that acute stress significantly reduced activation in channels CH15, CH38, and CH67, while significantly increasing activation in channel CH64 when athletes faced relatively unfair decisions.

To further explore the covariate relationship between decision-making behavior and brain function, the pre- and post-intervention behavioral differentials and brain activation differentials were examined using Spearman’s rank correlation analysis. The results showed that there was a moderate negative correlation between the difference in acceptance efficiency of the athletes before and after the acute stress and the CH38 brain activation difference (*r* = −0.425, *p* = 0.006) (see Figure 5B) and a moderate positive correlation with the CH64 brain activation difference (*r* = 0.499, *p* = 0.001) (see Figure 5D). These findings indicate that behavioral changes in athletes receiving relatively unfair treatment following acute stress are closely associated with reduced activation at CH38 and increased activation at CH64.

## 4 Discussion

This study employed a randomized controlled design integrating fNIRS, the UG, and the MAST to investigate the behavioral and neural correlates of unfairness-related decision-making under acute stress. Specifically, we examined prefrontal and bilateral temporoparietal cortical hemodynamic responses in athletes and non-athletes following stress induction. The results demonstrated that acute stress significantly modulated unfairness-related decision-making. Compared with non-athletes, athletes were more likely to accept relatively unfair offers after stress exposure. This behavioral tendency was associated with reduced activation in channel 38 (supramarginal gyrus, Brodmann area 40) and increased activation in channel 64 (primary motor cortex, Brodmann area 4).

The present study found no significant differences between athletes and non-athletes in unfairness-related decision-making under baseline conditions. We hypothesize that such decisions may be influenced by social norms and environmental context. Although the recruited student-athletes had undergone prolonged high-intensity professional training prior to university, they share the same campus culture, educational environment, and social context as their ordinary peers. Consequently, their core values, behavioral patterns, and fundamental perceptions of fairness are likely comparable to those of non-athletes. However, following acute stress induction, a significant divergence emerged: athletes exhibited a greater propensity to accept relatively unfair offers compared with non-athletes. As a distinct population, athletes routinely face high-intensity training and competitive pressures, encountering stressors less common among their peers, such as injuries and performance setbacks. Prior research supports the notion that prolonged exposure to competitive environments shapes adaptive decision-making strategies. For example, Krohne and Hindel (2000) reported that successful collegiate table tennis players frequently employed avoidance strategies during matches. Similarly, Gaudreau et al. (2002) and Gaudreau and Blondin (2004) found that task-oriented coping facilitated goal achievement, enhanced psychological adjustment, and improved decision-making among elite golfers. These findings suggest that sustained training and competition foster the ability to rapidly adjust strategies under pressure to optimize outcomes. Consistent with these observations, the present study indicates that athletes adaptively modulate their unfairness-related decisions under acute stress, demonstrating higher tolerance toward relatively unfair offers. This behavior appears to reflect a learned, proactive strategy rather than cognitive depletion, akin to tactical compromise in competitive settings, potentially aimed at achieving short-term goals or maintaining performance. Such adaptive decision-making may confer advantages not only in athletic contexts but also in daily life and career development. In contrast, non-athletes, lacking comparable training and psychological adaptation experience, appear less capable of adjusting their decisions effectively under stress. These findings provide important insights into group differences in behavioral and psychological responses to acute stress and offer valuable implications for elucidating the mechanisms through which stress influences decision-making.

Knoch et al. (2008) demonstrated that transcranial direct current stimulation (tDCS) inhibition of the right dorsolateral prefrontal cortex (dlPFC) increased participants’ acceptance of unfair offers, highlighting the causal role of specific brain region activity in fairness-related decision-making. Consistently, the present study found that following acute stress, athletes exhibited decreased activation in CH38 (supramarginal gyrus, Brodmann area 40) and increased activation in CH64 (primary motor cortex, M1, Brodmann area 4), which correlated with their greater acceptance of relatively unfair offers. The supramarginal gyrus plays a critical role in social cognition, emotion regulation, and decision-making; reduced activation in this region under stress may diminish sensitivity to unfair information, attenuate excessive fairness evaluation, and allow athletes to focus more on the feasibility of their actions, thereby influencing decision outcomes. The concomitant increase in M1 activation may reflect enhanced physiological arousal and action preparation under stress, enabling athletes to integrate relevant information rapidly and respond more efficiently during decision-making tasks. These neural dynamics suggest that the observed decision-making advantage in athletes under stress is not incidental but likely represents a biological adaptation resulting from prolonged training and competitive experience. Parallel to Knoch et al.’s (2008) findings, our results emphasize the importance of activity modulation in specific brain regions during fairness-related decisions, where reduced sensitivity to unfairness facilitates more flexible behavioral responses. Notably, the elevated M1 activation further indicates that stress-induced decision-making in athletes involves not only cognition- and emotion-related regions (e.g., supramarginal gyrus) but also regions associated with motor preparation and physiological readiness, highlighting a coordinated multi-regional neural mechanism. Li and Smith (2021), in a systematic review of the neural efficiency hypothesis, reported that prolonged specialized training induces neuroplasticity and functional optimization in athletes brains, resulting in greater neural efficiency. In this study, athletes under stress conditions, when facing relatively unfair decisions, exhibited a neural activity pattern characterized by decreased activation in CH38 (supramarginal gyrus) and increased activation in CH64 (primary motor cortex). This pattern indicates a reduced reliance on brain areas related to cognition and emotion, while enhancing the activity in regions associated with behavioral execution. Such optimization of neural resource allocation aligns closely with the core principles of the neural efficiency hypothesis, providing support for the changes in the neural mechanisms underlying athletes’ decision-making behavior under stress in this study.

Despite demonstrating the significant impact of acute stress on athletes’ fairness-related decision-making and its association with specific neural activity, several limitations should be acknowledged. First, this study focused solely on the immediate effects of acute stress, without tracking the dynamic changes in behavior and brain function during the recovery period. Future research could adopt a multiple time-point design (e.g., 0, 30, and 60 min post-stress) to elucidate the temporal dynamics and recovery mechanisms of stress effects. Second, the study did not account for potential differences in cold pressor pain tolerance between athletes and non-athletes (Wakabayashi et al., 2025) and failed to incorporate subjective stress measurement tools (e.g., SAM or STAI-6). This may have obscured differences between objective and subjective stress responses, particularly in athletes, whose high pain tolerance might result in lower subjective stress perception. Future research should integrate both subjective and objective measurements and include controls for pain sensitivity. Third, menstrual cycle information was not collected during the experimental design phase, preventing analysis of the cycle distribution of female participants or statistical control for hormonal influences. Research indicates that menstrual cycles and fluctuations in estrogen and progesterone can significantly affect prefrontal cortex blood oxygen signals and decision-making preferences (Chung et al., 2016; Senior et al., 2007). Future studies should collect information on the last menstrual period or salivary hormones (estradiol, progesterone) on the experiment day to systematically validate the generalizability of findings. Fourth, although blood pressure was employed as an index of acute stress, measuring salivary cortisol would provide direct evidence of HPA axis activation. Finally, the relatively modest sample size of athletes in this study may have limited statistical power, contributing to several effects approaching, but not reaching, significance. Increasing the sample size in future studies could enhance the robustness and generalizability of the findings.

## 5 Conclusion

The study found that athletes tend to accept relatively unfair solutions after acute stress and that this behavioral performance is associated with reduced activation in the supramarginal gyrus and elevated activation in the primary motor cortex when athletes face relatively unfair proposals under acute stress. This study provides new perspectives for understanding the psychological and neural mechanisms of athletes in stressful situations, and also provides important theoretical references for athletes’ psychological training and competition strategy development.

## Data Availability

The raw data supporting the conclusions of this article will be made available by the authors, without undue reservation.
