# Supplementary material for: The impact of acute stress on athletes’ perceptions of fairness in decision-making and its neural mechanisms
Source: Front Hum Neurosci. 2025 Oct 30;19:1685000. doi: 10.3389/fnhum.2025.1685000 (PMC12611808; doi:10.3389/fnhum.2025.1685000)
Supplement: Supplementary file 1 [file Data_Sheet_1.docx]

**Supplementary Materials**

**1 Blood pressure and behavior data results**

Table 1 Descriptive statistics for blood pressure and heart rate (M±SD)

| Blood pressure | Time | Athlete stress group | Athlete sedentary group | Ordinary person stress group | Ordinary person sedentary group |
| --- | --- | --- | --- | --- | --- |
| Diastolic blood pressure (mmHg) | Pre-test | 71.0±4.522 | 68.7±5.908 | 72.9±6.027 | 70.3±4.923 |
|  | Post-test | 73.9±7.695 | 76.0±11.225 | 76.0±11.225 | 69.9±10.979 |
| Systolic blood pressure (mmHg) | Pre-test | 114.0±11.804 | 109.2±9.784 | 113.8±4.826 | 109.6±7.590 |
|  | Post-test | 123.4±12.465 | 119.5±12.678 | 119.5±12.678 | 110.0±10.424 |
| Heart rate (PM) | Pre-test | 73.8±14.459 | 70.1±10.619 | 78.0±9.475 | 69.2±9.355 |
|  | Post-test | 73.8±12.761 | 73.1±6.9 | 77.3±11.196 | 76.3±9.8 |


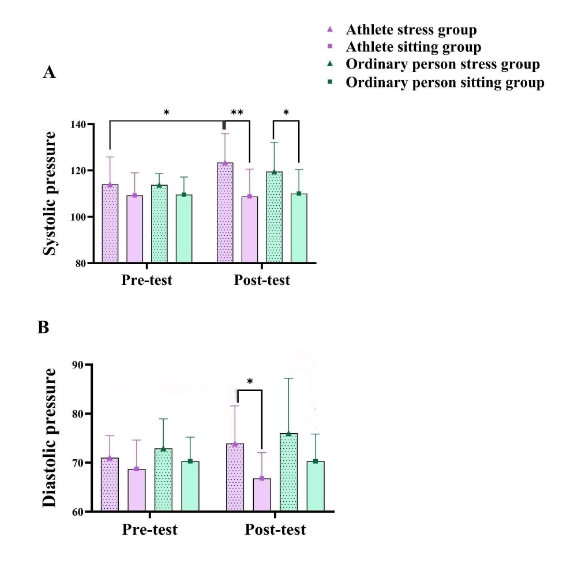


Figure 1 Between-group and pre- and post-comparison results of systolic and diastolic blood pressure

Table 2 Behavioural descriptive statistics results (M±SD)

|  |  | Group | Pre-test | | Post-test | |
| --- | --- | --- | --- | --- | --- | --- |
|  |  |  | Mean value | Standard deviation | Mean value | Standard deviation |
| C1 | Rejecting Efficiency | 1 | 0.05 | 0.07 | 0.11 | 0.07 |
|  |  | 2 | 0.04 | 0.05 | 0.07 | 0.07 |
|  |  | 3 | 0.05 | 0.05 | 0.11 | 0.07 |
|  |  | 4 | 0.08 | 0.07 | 0.13 | 0.08 |
|  | Acceptance efficiency | 1 | 0.02 | 0.03 | 0.03 | 0.07 |
|  |  | 2 | 0.02 | 0.03 | 0.06 | 0.09 |
|  |  | 3 | 0.01 | 0.02 | 0.01 | 0.03 |
|  |  | 4 | 0.01 | 0.03 | 0.02 | 0.04 |
| C2 | Rejecting Efficiency | 1 | 0.01 | 0.02 | 0.01 | 0.03 |
|  |  | 2 | 0.01 | 0.01 | 0.02 | 0.04 |
|  |  | 3 | 0.03 | 0.03 | 0.06 | 0.06 |
|  |  | 4 | 0.04 | 0.04 | 0.14 | 0.10 |
|  | Acceptance efficiency | 1 | 0.07 | 0.02 | 0.17 | 0.08 |
|  |  | 2 | 0.07 | 0.08 | 0.10 | 0.08 |
|  |  | 3 | 0.03 | 0.03 | 0.04 | 0.04 |
|  |  | 4 | 0.05 | 0.05 | 0.05 | 0.06 |
| C3 | Rejecting Efficiency | 1 | 0.00 | 0.01 | 0.00 | 0.00 |
|  |  | 2 | 0.01 | 0.01 | 0.00 | 0.00 |
|  |  | 3 | 0.01 | 0.03 | 0.01 | 0.02 |
|  |  | 4 | 0.03 | 0.05 | 0.04 | 0.10 |
|  | Acceptance efficiency | 1 | 0.11 | 0.07 | 0.17 | 0.05 |
|  |  | 2 | 0.06 | 0.04 | 0.12 | 0.07 |
|  |  | 3 | 0.06 | 0.04 | 0.12 | 0.05 |
|  |  | 4 | 0.07 | 0.05 | 0.15 | 0.08 |

Note: C1:extremely unfair scenario, C2:relatively unfair scenario, C3:absolutely fair scenario.

Table 3 Behavioural repeated measures ANOVA results

|  |  | F | *p* | *η*2 |
| --- | --- | --- | --- | --- |
| Time | C1 Rejection Efficiency | 16.90 | 0.000 | 0.32 |
|  | C1 acceptance efficiency | 1.74 | 0.196 | 0.05 |
|  | C2 Rejection Efficiency | 17.48 | 0.000 | 0.33 |
|  | C2 acceptance efficiency | 9.16 | 0.005 | 0.20 |
|  | C3 Rejection Efficiency | 0.00 | 1.000 | 0.00 |
|  | C3 acceptance efficiency | 33.50 | 0.000 | 0.48 |
| Time*Group | C1 Rejection Efficiency | 0.30 | 0.827 | 0.02 |
|  | C1 acceptance efficiency | 0.46 | 0.711 | 0.04 |
|  | C2 Rejection Efficiency | 6.16 | 0.002 | 0.34 |
|  | C2 acceptance efficiency | 3.25 | 0.033 | 0.21 |
|  | C3 Rejection Efficiency | 0.74 | 0.534 | 0.06 |
|  | C3 acceptance efficiency | 0.30 | 0.823 | 0.03 |
| Group | C1 Rejection Efficiency | 1.68 | 0.190 | 0.12 |
|  | C1 acceptance efficiency | 1.26 | 0.303 | 0.10 |
|  | C2 Rejection Efficiency | 7.80 | 0.000 | 0.39 |
|  | C2 acceptance efficiency | 7.31 | 0.001 | 0.38 |
|  | C3 Rejection Efficiency | 1.31 | 0.287 | 0.10 |
|  | C3 acceptance efficiency | 2.51 | 0.075 | 0.17 |

Table 4 ANOVA results of changes in brain area channels before and after stress in different groups

| Effective volume | Channel | *F* | *p* | *η^2^* |
| --- | --- | --- | --- | --- |
| Group × Condition  Interaction effect | CH6 | 4.111 | 0.046 | 0.054 |
|  | CH7 | 5.897 | 0.018 | 0.076 |
|  | CH22 | 7.772 | 0.007 | 0.097 |
| Group × pre-post test  Interaction Effect | CH13 | 6.57 | 0.012 | 0.084 |
|  | CH38 | 5.014 | 0.028 | 0.065 |
|  | CH67 | 4.62 | 0.035 | 0.06 |
| Condition × pre-post test  Interaction Effect | CH32 | 5.523 | 0.022 | 0.071 |
|  | CH38 | 4.634 | 0.035 | 0.06 |
|  | CH54 | 5.421 | 0.023 | 0.07 |
|  | CH58 | 6.861 | 0.011 | 0.087 |
|  | CH64 | 9.803 | 0.003 | 0.12 |
| Group × condition × pre-posttest interaction effect | CH5 | 4.035 | 0.048 | 0.053 |
|  | CH15 | 6.776 | 0.011 | 0.086 |

Table 5 ANOVA results of acute stress on changes in channels in brain regions receiving relative inequity

| Effective volume | Channel | *F* | *p* | *η^2^* |
| --- | --- | --- | --- | --- |
| Group x time  Interaction effect | CH38 | 5.014 | 0.028 | 0.065 |
|  | CH67 | 4.62 | 0.035 | 0.060 |
| Condition × Time  Interaction effect | CH38 | 4.634 | 0.035 | 0.06 |
|  | CH64 | 9.803 | 0.003 | 0.120 |
| Group × condition × time interaction effect | CH15 | 6.776 | 0.011 | 0.086 |

**2. Localisation of brain regions**

Table 6 Localisation of Brudermann's brain regions

| Channels | Brodmann Area | X | Y | Z |
| --- | --- | --- | --- | --- |
| CH01 | 10 - Frontopolar area | 46 | 53 | 5 |
| CH02 | 10 - Frontopolar area | 26 | 67 | 17 |
| CH03 | 10 - Frontopolar area | 9 | 72 | 17 |
| CH04 | 10 - Frontopolar area | 36 | 62 | 17 |
| CH05 | 46 - Dorsolateral prefrontal cortex | 53 | 39 | 13 |
| CH06 | 10 - Frontopolar area | 39 | 56 | 22 |
| CH07 | 10 - Frontopolar area | 10 | 66 | 29 |
| CH08 | 10 - Frontopolar area | 22 | 62 | 31 |
| CH09 | 46 - Dorsolateral prefrontal cortex | 48 | 47 | 24 |
| CH10 | 46 - Dorsolateral prefrontal cortex | 47 | 42 | 28 |
| CH11 | 46 - Dorsolateral prefrontal cortex | 24 | 54 | 39 |
| CH12 | 46 - Dorsolateral prefrontal cortex | 8 | 56 | 44 |
| CH13 | 46 - Dorsolateral prefrontal cortex | 35 | 48 | 39 |
| CH14 | 46 - Dorsolateral prefrontal cortex | 54 | 20 | 34 |
| CH15 | 8 - Includes Frontal eye fields | 37 | 33 | 47 |
| CH16 | 8 - Includes Frontal eye fields | 9 | 43 | 55 |
| CH17 | 8 - Includes Frontal eye fields | 17 | 41 | 55 |
| CH18 | 8 - Includes Frontal eye fields | 42 | 30 | 49 |
| CH19 | 8 - Includes Frontal eye fields | 46 | 16 | 52 |
| CH20 | 8 - Includes Frontal eye fields | 24 | 30 | 59 |
| CH21 | 8 - Includes Frontal eye fields | 2 | 32 | 59 |
| CH22 | 8 - Includes Frontal eye fields | 29 | 23 | 62 |
| CH23 | 21 - Middle Temporal gyrus | 66 | 56 | 0 |
| CH24 | 22 - Superior Temporal Gyrus | 69 | 42 | 15 |
| CH25 | 2 - Primary Somatosensory Cortex x | 68 | 20 | 31 |
| CH26 | 37 - Fusiform gyrus | 59 | 71 | 4 |
| CH27 | 22 - Superior Temporal Gyrus | 66 | 55 | 16 |
| CH28 | 40 - Supramarginal gyrus part of Wernicke’s area | 67 | 38 | 35 |
| CH29 | 2 - Primary Somatosensory Cortex | 65 | 19 | 40 |
| CH30 | 39 - Angular gyrus, part of Wernicke’s area | 59 | 68 | 19 |
| CH31 | 40 - Supramarginal gyrus part of Wernicke’s area | 63 | 52 | 35 |
| CH32 | 40 - Supramarginal gyrus part of Wernicke’s area | 64 | 34 | 46 |
| CH33 | 19 - V3 | 48 | 83 | 23 |
| CH34 | 39 - Angular gyrus, part of Wernicke’s area | 56 | 67 | 37 |
| CH35 | 40 - Supramarginal gyrus part of Wernicke’s area | 59 | 50 | 49 |
| CH36 | 2 - Primary Somatosensory Cortex | 54 | 28 | 58 |
| CH37 | 39 - Angular gyrus, part of Wernicke’s area | 48 | 78 | 37 |
| CH38 | 40 - Supramarginal gyrus part of Wernicke’s area | 53 | 63 | 49 |
| CH39 | 40 - Supramarginal gyrus part of Wernicke’s area | 49 | 46 | 59 |
| CH40 | 19 - V3 | 36 | 88 | 35 |
| CH41 | 19 - V3 | 42 | 76 | 46 |
| CH42 | 7 - Somatosensory Association Cortex | 41 | 59 | 60 |
| CH43 | 2 - Primary Somatosensory Cortex | 37 | 40 | 70 |
| CH44 | 19 - V3 | 33 | 85 | 44 |
| CH45 | 7 - Somatosensory Association Cortex | 33 | 70 | 59 |
| CH46 | 7 - Somatosensory Association Cortex | 30 | 54 | 72 |
| CH47 | 40 - Supramarginal gyrus part of Wernicke’s area | 68 | 28 | 43 |
| CH48 | 40 - Supramarginal gyrus part of Wernicke’s area | 65 | 48 | 36 |
| CH49 | 39 - Angular gyrus, part of Wernicke’s area | 58 | 69 | 26 |
| CH50 | 2 - Primary Somatosensory Cortex | 60 | 22 | 53 |
| CH51 | 40 - Supramarginal gyrus part of Wernicke’s area | 62 | 44 | 50 |
| CH52 | 40 - Supramarginal gyrus part of Wernicke’s area | 56 | 63 | 42 |
| CH53 | 19 - V3 | 47 | 81 | 29 |
| CH54 | 40 - Supramarginal gyrus part of Wernicke’s area | 55 | 35 | 58 |
| CH55 | 40 - Supramarginal gyrus part of Wernicke’s area | 55 | 55 | 53 |
| CH56 | 39 - Angular gyrus, part of Wernicke’s area | 48 | 73 | 45 |
| CH57 | 3 - Primary Somatosensory Cortex | 45 | 26 | 68 |
| CH58 | 40 - Supramarginal gyrus part of Wernicke’s area | 43 | 49 | 64 |
| CH59 | 7 - Somatosensory Association Cortex | 40 | 66 | 58 |
| CH60 | 19 - V3 | 34 | 81 | 47 |
| CH61 | 7 - Somatosensory Association Cortex | 35 | 41 | 72 |
| CH62 | 7 - Somatosensory Association Cortex | 33 | 59 | 69 |
| CH63 | 7 - Somatosensory Association Cortex | 28 | 75 | 58 |
| CH64 | 4 - Primary Motor Cortex | 21 | 34 | 78 |
| CH65 | 7 - Somatosensory Association Cortex | 25 | 53 | 74 |
| CH66 | 7 - Somatosensory Association Cortex | 23 | 66 | 69 |
| CH67 | 7 - Somatosensory Association Cortex | 19 | 80 | 56 |
| CH68 | 7 - Somatosensory Association Cortex | 15 | 42 | 80 |
| CH69 | 7 - Somatosensory Association Cortex | 19 | 61 | 73 |
| CH70 | 7 - Somatosensory Association Cortex | 15 | 74 | 64 |
